# Socioeconomic Differences in Navigating Access to Lung Transplant

**DOI:** 10.1001/jamanetworkopen.2025.0572

**Published:** 2025-03-13

**Authors:** Carli J. Lehr, Lyla Mourany, Paul Gunsalus, Johnie Rose, Maryam Valapour, Jarrod E. Dalton

**Affiliations:** 1 Department of Pulmonary Medicine, Integrated Hospital Care Institute, Cleveland Clinic, Cleveland, Ohio; 2 Cleveland Clinic Lerner College of Medicine of Case Western Reserve University, Cleveland, Ohio; 3 Center for Populations Health Research, Department of Quantitative Health Sciences, Lerner Research Institute, Cleveland Clinic, Cleveland, Ohio; 4 Center for Community Health Integration, Case Western Reserve University School of Medicine, Cleveland, Ohio

## Abstract

**Importance:**

Inequitable access to transplant in the US is well recognized, yet the nature and extent of upstream disparities in care prior to transplant are unknown.

**Objective:**

To understand patterns of referral for lung transplant by race, ethnicity, and neighborhood-level socioeconomic status.

**Design, Setting, and Participants:**

This retrospective cohort study included adults aged 18 to 80 years with obstructive and restrictive lung disease from a single large-volume transplant center in Cleveland, Ohio, who were diagnosed between January 1, 2006, and May 11, 2023.

**Exposures:**

Neighborhood resources.

**Main Outcomes and Measures:**

The main outcome was the transition to the next stage of the transplant care continuum, death, or a lapse in care. Cause-specific Cox proportional hazards regression models were used to account for death as a competing risk, adjusting for age at index encounter (respective to each cohort), diagnosis, and sex as covariates.

**Results:**

This study included 30 050 patients with obstructive and restrictive lung disease with primary care encounters (mean [SD] age, 65 [13] years; 56.1% female), 73 817 with a pulmonary medicine encounter, 4198 undergoing lung transplant evaluation, and 1378 on the lung transplant waiting list. In a multivariable model including age, diagnosis, sex, area deprivation index, and race and ethnicity (including 3.3% Hispanic, 15.2% non-Hispanic Black, and 81.5% non-Hispanic White individuals), patients residing in the least-resourced neighborhoods were 97% more likely to die without transitioning to pulmonary medicine (hazard ratio [HR], 1.97 [95% CI, 1.78-2.17]), 90% more likely to die prior to lung transplant evaluation (HR, 1.90 [95% CI, 1.77-2.04]), 40% more likely to die prior to placement on the waiting list (HR, 1.40 [95% CI, 1.11-1.76]), and 97% more likely to die prior to transplant (HR, 1.97 [95% CI, 1.18-3.29]) compared with patients residing in the most-resourced neighborhoods. These patients were also 13% less likely to transition to pulmonary medicine (HR, 0.87 [95% CI, 0.82-0.92]) and 45% less likely to be placed on the waiting list (HR, 0.55 [95% CI, 0.44-0.68]) despite a 69% increased likelihood of transplant evaluation (HR, 1.69 [95% CI, 1.36-2.09]). While non-Hispanic Black patients had lower risks of death across all stages of care, they experienced a 39% lower likelihood of proceeding to lung transplant evaluation (HR, 0.61 [95% CI, 0.51-0.74]). Racial differences in the cumulative incidence of waiting list placement were found, but differences were not consistent across levels of neighborhood resources.

**Conclusions and Relevance:**

In this retrospective cohort study of patients diagnosed with restrictive and obstructive pulmonary disease, increased mortality risks and decreased likelihood of care escalations for patients who were socioeconomically disadvantaged and for racial and ethnic minority patients were found. These results suggest potential interventions for advancing equitable access to lung transplant.

## Key Points

**Question:**

Are patient demographics and socioeconomic status associated with access to the US lung transplant system?

**Findings:**

In this cohort study of patients with obstructive and restrictive lung disease, patients in the least-resourced neighborhoods were more likely to die in each stage of the transplant care continuum. Despite a 69% increased likelihood of transplant evaluation, they were 45% less likely to be placed on the waiting list but experienced similar transplant rates.

**Meaning:**

The findings of this study suggest that lower socioeconomic status may be an upstream barrier to accessing lung transplant care.

## Teaser

This cohort study assesses whether patient demographics and socioeconomic status are associated with access to the lung transplant system.

## Introduction

In the US, an individual’s likelihood of referral for organ transplant, eventual placement on the transplant waiting list, and receipt of an organ transplant varies significantly based on race and ethnicity, sex, geographic location, and socioeconomic status (SES).^1^ Social determinants of health have been shown to be factors in the likelihood of patient evaluation and access to transplant. Disparities in access have been demonstrated at multiple time points in the transplant process for individuals with a lower SES as well as for racial and ethnic minorities.^2,3,4^ Disparities in upstream referral patterns have been well-described in kidney transplantation through analyses of the US Renal Data System,^2,3,4,5^ but such evidence in lung transplantation is more scarce due to the lack of systematic data prior to wait-listing.

This lack of data makes it difficult to address disparities in access to transplant for individuals with advanced lung disease. We hypothesized that an individual’s likelihood of successfully navigating the lung transplant care continuum is associated with not only their SES but also with nonmodifiable factors such as race and ethnicity. This study aims to systematically evaluate socioeconomic, racial, and ethnic disparities in patients’ likelihood of transition through different points in medical care and risk of mortality.

## Methods

Ethics approval for this cohort study was obtained from the Institutional Review Board of the Cleveland Clinic Healthcare System (CCHS); a waiver of informed consent was granted because of the presence of minimal risk and the use of deidentified data. The study followed the Strengthening the Reporting of Observational Studies in Epidemiology (STROBE) reporting guideline.

### Participants

Participants were identified from electronic health records (EHRs) from a single large-volume transplant center in Cleveland, Ohio, from January 1, 2006, to May 11, 2023. Individuals who are US lung transplant candidates are characterized into 4 diagnosis groups: obstructive lung disease (eg, chronic obstructive pulmonary disease), pulmonary vascular disease, cystic fibrosis and immunodeficiency disorders, and restrictive lung disease (eg, idiopathic pulmonary fibrosis).^6^ The cohort was limited to adults aged 18 to 80 years at the time of diagnosis with obstructive and restrictive lung disease using *International Classification of Diseases, Ninth Revision* (*ICD-9*) and *International Statistical Classification of Diseases and Related Health Problems, Tenth Revision* (*ICD-10*) codes (eTable 1 in Supplement 1), as 99% of lung transplant recipients are in this age group, and 92% have obstructive or restrictive lung disease.^7^ We included patients whose self-reported race and ethnicity were Hispanic, non-Hispanic Black (hereinafter Black), and non-Hispanic White (hereinafter White). Other racial and ethnic groups could not be evaluated with sufficient precision due to sample-size limitations. Patients were excluded (3% to 7% across cohorts) if their location of residence could not be geocoded or associated with measures of neighborhood SES.

### Study Variables

Primary care (cohort 1) was defined as a practitioner or department specialty of internal medicine, family medicine, emergency medicine, obstetrics and gynecology, or geriatrics within the CCHS. Pulmonary medicine (cohort 2) was defined as a practitioner or department specialty of pulmonary medicine, excluding a practitioner or department specialty of pulmonary transplant. Lung transplant evaluation (cohort 3) was defined using a practitioner or department specialty of pulmonary transplant. Waiting list and transplant (cohort 4) were defined by the presence of data within the EHR and cross-referenced with data submitted directly to the Organ Procurement and Transplantation Network (OPTN) within transplant-specific modules located in the EHR (eTable 2 in Supplement 1).

Neighborhood SES was characterized using the 2017 to 2021 area deprivation index (ADI) at the US census block-group level for a patient’s home residence (quintile 1: most resources; quintile 5: least resources)^8^ using the sociome package^9^ in R, version 2.2.5 (R Project for Statistical Computing). We reestimated national ADI values to address a known limitation of other ADI software.^10^ Other study variables included comorbid illnesses as characterized using *ICD-9* or *ICD-10* codes and risk factors included in US lung allocation mortality risk models (eTable 3 in Supplement 1).

### Study Design

This study involved the analysis of 4 retrospective observational cohorts. Cohort 1 began at the first encounter in primary care and required 2 encounters within a 2-year period to reduce bias from underdiagnosis of lung disease. Cohort 2 began at the initial encounter in pulmonary medicine. Cohort 3 began at the initial encounter for transplant evaluation, and cohort 4 began at placement on the waiting list. Time-to-event outcomes included time to encounter at the next level of care (or transplant for cohort 4); time to lapse in care (defined as the start to end date of any contiguous 2-year period without encounters at the current level of care [lost to follow-up]), and time to death. Cohort 1 included patients who resided in Ohio at study entry (as out-of-state patients were unlikely to have a primary care practitioner within the CCHS), while cohorts 2 to 4 included patients residing anywhere in the US, owing to the CCHS’s status as a specialty referral center. Death dates were obtained from the EHR using the Ohio Department of Health and the Social Security death master file. The time period for accrual into each of the cohorts was January 1, 2006, to February 28, 2023. Outcome data were censored if the patients’ last encounter was less than 2 years prior to February 28, 2023.

### Statistical Analysis

In our primary analysis, we studied the association between the ADI quintile and the transition between each cohort using cause-specific Cox proportional hazards regression models to account for death as a competing risk and adjusted for age at index encounter (respective to each cohort, as aforementioned), diagnosis, and sex as covariates. As a secondary analysis, we studied the interaction of the ADI and race and ethnicity to understand variation in the association between the ADI quintile and rates of transition to the next level of care by rates of transition to the next cohort by race and ethnicity. Finally, we incorporated a main effect for race and ethnicity as well as an interaction effect between the ADI and race and ethnicity into the aforementioned models. Additional biologic factors were not included as covariates, as they did not represent confounding variables in proposed associations explained by these models. Statistical significance was determined using *t* tests at a 2-sided *P* value < .05. CCHS EHR data are maintained using the Epic platform (Epic Systems Corp). We extracted study data using SQL Server Management Studio (2019) (Microsoft Corp). Statistical analysis was performed using R, version 4.3.1; Posit Workbench (Posit Software); and RStudio Pro, version 2023.09.0 (Posit, PBC).^11^

## Results

This study included 30 050 patients with obstructive and restrictive lung disease with primary care encounters (mean [SD] age, 65 [13] years; 56.1% female and 43.9% male) of whom 94.9% were with obstructive lung disease, 73 817 with a pulmonary medicine encounter (78.1% with obstructive lung disease), 4198 undergoing lung transplant evaluation (49.7% with obstructive lung disease), and 1378 on the lung transplant waiting list (39.6% with obstructive lung disease). Among all patients, 15.2% were Black, 3.3% were Hispanic, and 81.5% were White.

### Cohort 1: Primary Care Patients With Obstructive or Restrictive Lung Disease

**Figure 1.**
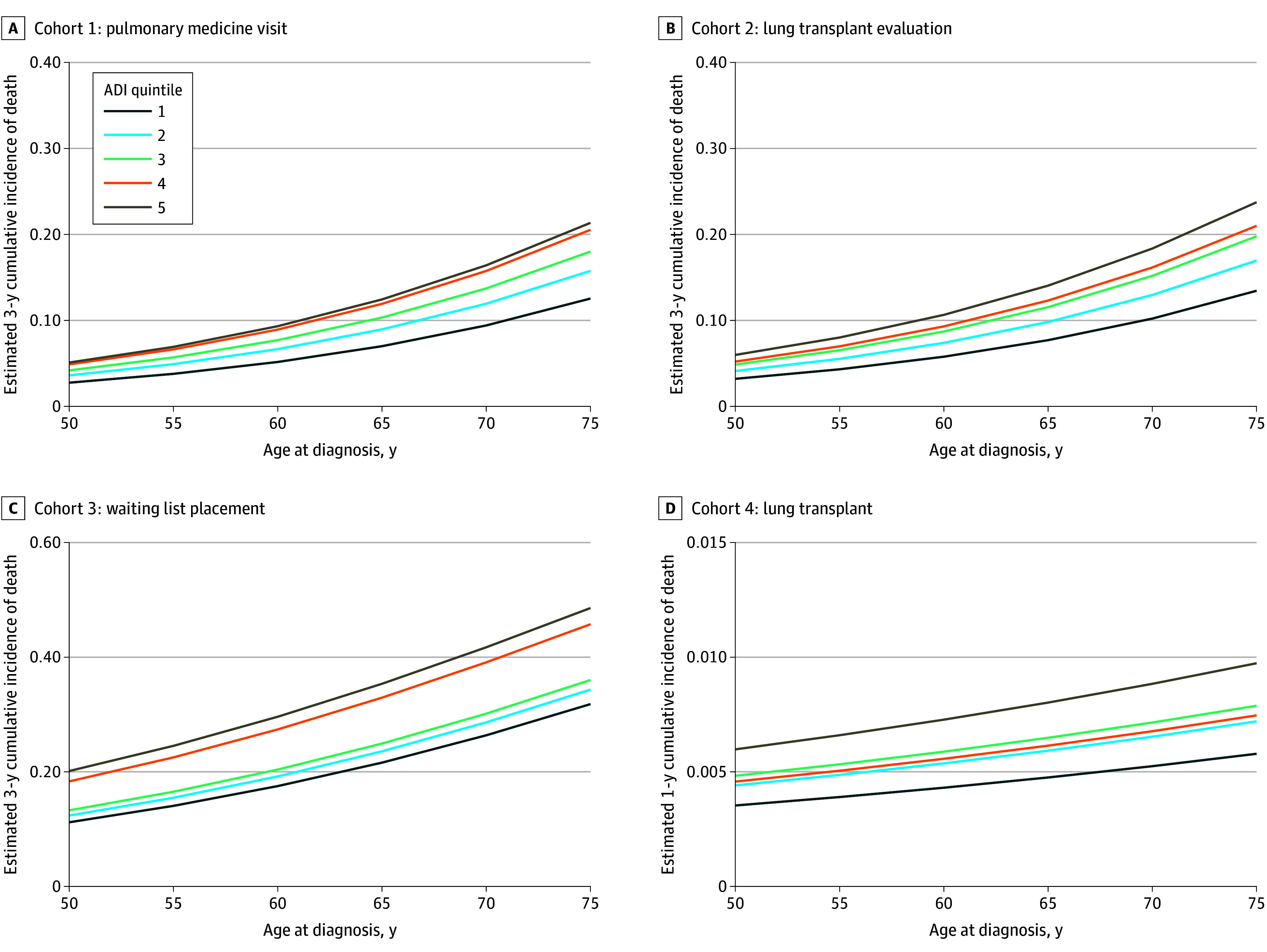
Cumulative Incidence of Death Across the Lung Transplant Care Continuum by Socioeconomic Status Model-estimated 3-year cumulative incidence of death prior to transitioning to the next level of care for primary care to pulmonary medicine (A), pulmonary medicine to lung transplant evaluation (B), and lung transplant evaluation to waiting list (C). D, Model-estimated 1-year cumulative incidence of death prior to transitioning to lung transplant for waiting list to lung transplant. Patients in the Cleveland Clinic Healthcare System were grouped according to area deprivation index (ADI) quintiles (quintile 1: most resources; quintile 5: least resources)^8^ associated with place of residence. Estimates are provided across age at diagnosis and the ADI for a male patient with restrictive lung disease. (Estimates for a male patient with obstructive lung disease are provided in eFigure 3 in Supplement 1.)

**Figure 2.**
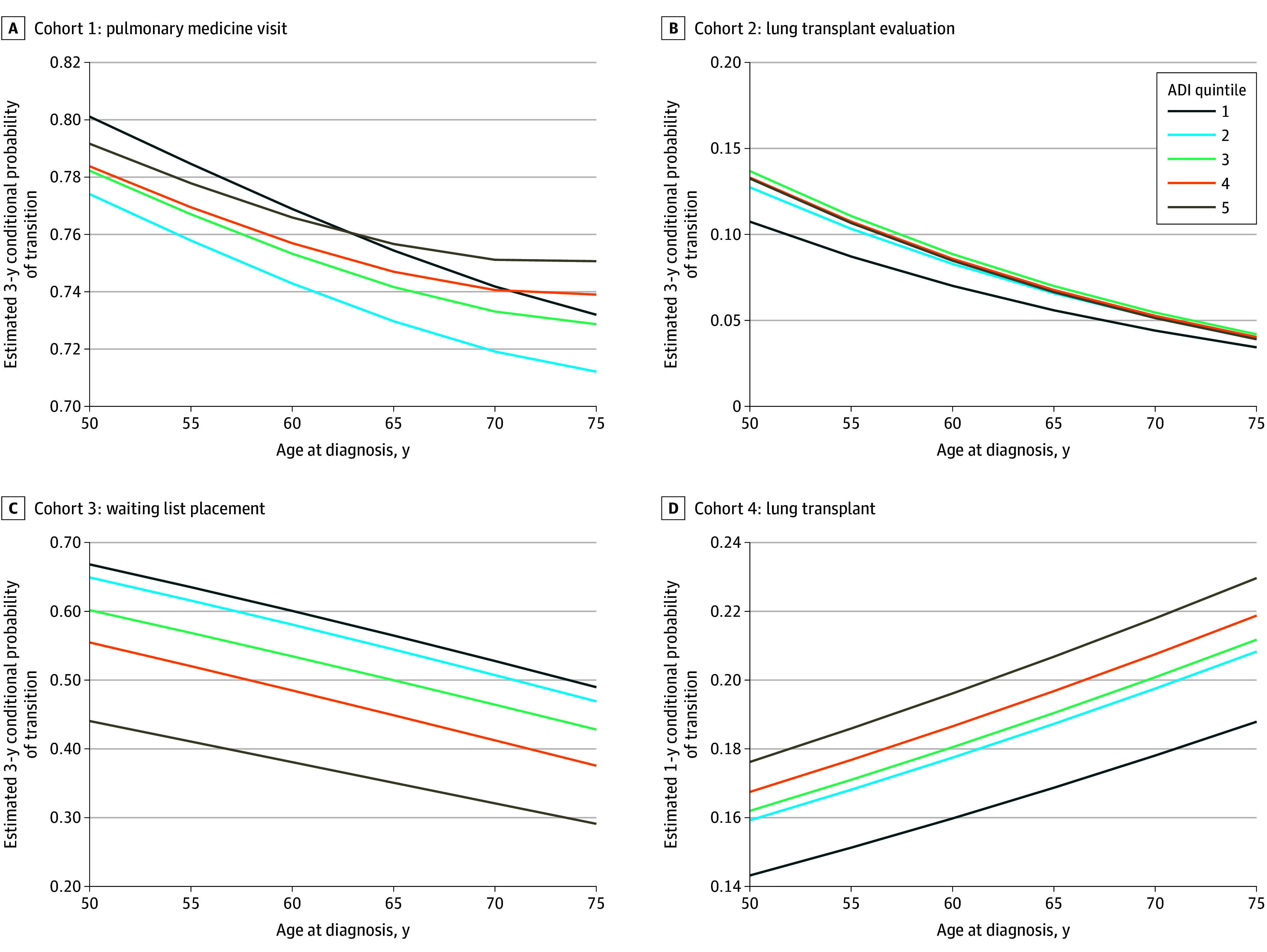
Conditional Probability of Transition Across the Lung Transplant Care Continuum by Socioeconomic Status Three-year conditional probability of transition between primary care to pulmonary medicine (A), pulmonary medicine to lung transplant evaluation (B), and lung transplant evaluation to waiting list (C). D, One-year conditional probability of transition from waiting list to lung transplant. Estimates are provided across age at diagnosis and area deprivation index (ADI) quintiles (quintile 1: most resources; quintile 5: least resources)^8^ and report the probability of transition assuming patients did not die during the period for a male patient with restrictive lung disease. (Estimates for a male patient with obstructive lung disease are provided in eFigure 4 in Supplement 1.)

There were 30 050 patients with a primary residence in Ohio who were diagnosed with obstructive (94.9%) or restrictive (5.1%) lung disease and who had at least 2 primary care encounters during the study period (Table 1 and eFigure 1 in Supplement 1). This cohort was 56.1% female and balanced across ADI quintiles. Among the patients in this cohort, 2774 (9.2%) experienced a lapse in care of more than 2 years, 6513 (21.7%) died, 17 236 (57.4%) transitioned to pulmonary medicine, and 3527 (11.7%) were censored for not enough follow-up time. Patients in the least-resourced ADI quintile (quintile 5) were 97% more likely to die before accessing pulmonary medicine (hazard ratio [HR], 1.97 [95% CI, 1.78-2.17]) compared with those in the most-resourced ADI quintile (quintile 1) while experiencing a 55% higher likelihood of lapsing (HR, 1.55 [95% CI, 1.33-1.81]) and a 13% lower rate of progressing to pulmonary medicine (HR, 0.87 [95% CI, 0.82-0.92]) (Figure 1A). Dose-response was observed, with increasing mortality risk across ADI quintiles (Table 2). Black patients experienced a lower risk of mortality (HR, 0.83 [95% CI, 0.77-0.89]) but were 19% more likely to transition to pulmonary medicine (HR, 1.19 [95% CI, 1.15-1.24]) compared with White patients (Table 2). Hispanic patients experienced a similar risk of mortality, lapse, and transition to pulmonary medicine. Those with restrictive lung disease were 30% less likely to experience a lapse in care (HR, 0.70 [95% CI, 0.54-0.91]) and were 86% more likely to successfully transition to pulmonary medicine (HR, 1.86 [95% CI, 1.74-1.98]) (Figure 2A). The cumulative incidence of lapse and cumulative incidence of death were highest for the oldest patients and those in the least-resourced ADI quintile (quintile 5) (Figure 1A and eFigure 2A in Supplement 1). Predictions for patients with obstructive lung disease are found in eFigures 3-5 in Supplement 1.

**Table 1.** Characteristics of Adult Patients With Obstructive and Restrictive Lung Disease by Sex and Cohort^a^

Characteristic	Patient sex								
	Female				Male				
	Cohort 1: primary care	Cohort 2: pulmonary medicine	Cohort 3: transplant evaluation	Cohort 4: waiting list	Cohort 1: primary care	Cohort 2: pulmonary medicine	Cohort 3: transplant evaluation	Cohort 4: waiting list
**Obstructive lung disease**									
Total, No.	16 047	31 778	1039	261	12 475	25 840	1047	285
Age at diagnosis, median (IQR), y	65 (56-74)	65 (56-74)	58 (52-63)	56 (47-60)	65 (57-74)	66 (58-74)	59 (51-65)	57 (47-63)
ADI quintile^b^								
1	1628 (10.0)	4839 (14.0)	109 (10.0)	34 (13.0)	1370 (11.0)	4262 (16.0)	135 (13.0)	46 (16.0)
2	3007 (19.0)	6771 (21.0)	227 (22.0)	69 (26.0)	2497 (20.0)	5810 (22.0)	240 (23.0)	68 (24.0)
3	3676 (23.0)	7634 (25.0)	304 (29.0)	69 (26.0)	3011 (24.0)	6498 (25.0)	269 (26.0)	79 (28.0)
4	3063 (19.0)	5669 (18.0)	208 (20.0)	51 (20.0)	2237 (18.0)	4328 (17.0)	198 (19.0)	52 (18.0)
5	4668 (29.0)	6865 (22.0)	191 (18.0)	38 (15.0)	3355 (27.0)	4942 (19.0)	205 (20.0)	40 (14.0)
Race and ethnicity								
Hispanic	288 (1.8)	1104 (3.5)	8 (0.8)	1 (0.4)	210 (1.7)	859 (3.3)	7 (0.7)	2 (0.7)
Non-Hispanic Black	3471 (22.0)	5216 (16.0)	87 (8.4)	16 (6.1)	2180 (17.0)	3184 (12.0)	104 (9.9)	24 (8.4)
Non-Hispanic White	12 288 (77.0)	25 458 (80.0)	944 (91.0)	244 (93.0)	10 085 (81.0)	21 797 (84.0)	936 (89.0)	259 (91.0)
**Restrictive lung disease**									
Total, No.	825	8859	771	271	703	7340	1341	561
Age at diagnosis, median (IQR), y	70 (59-80)	66 (55-74)	61 (53-67)	60 (53-65)	70 (61-77)	69 (59-76)	64 (57-69)	62 (57-67)
ADI quintile^b^								
1	165 (20.0)	1780 (20.0)	136 (18.0)	55 (20.0)	115 (16.0)	1632 (21.0)	254 (19.0)	106 (19.0)
2	190 (23.0)	2201 (25.0)	187 (24.0)	67 (25.0)	186 (26.0)	2003 (27.0)	372 (28.0)	173 (31.0)
3	188 (23.0)	2196 (25.0)	195 (25.0)	74 (27.0)	189 (27.0)	1809 (25.0)	355 (26.0)	152 (27.0)
4	119 (14.0)	1397 (16.0)	131 (17.0)	41 (15.0)	94 (13.0)	1068 (15.0)	219 (16.0)	87 (16.0)
5	163 (20.0)	1285 (15.0)	122 (16.0)	34 (13.0)	119 (17.0)	828 (11.0)	141 (11.0)	43 (7.7)
Race and ethnicity								
Hispanic	20 (2.4)	680 (7.7)	18 (2.3)	5 (1.8)	16 (2.3)	447 (6.1)	22 (1.6)	12 (2.1)
Non-Hispanic Black	174 (21.0)	1270 (14.0)	103 (13.0)	37 (14.0)	101 (14.0)	601 (8.2)	83 (6.3)	30 (5.5)
Non-Hispanic White	631 (76.0)	6909 (78.0)	650 (84.0)	229 (85.0)	586 (83.0)	6292 (86.0)	1236 (92.0)	519 (92.0)

Abbreviation: ADI, area deprivation index.

^a^ Data are presented as the No. (%) of patients unless otherwise indicated.

^b^ Quintile 1: most resources; quintile 5: least resources.^8^ Five female and 5 male patients with obstructive lung disease were missing an ADI quintile.

**Table 2.** Cox Proportional Hazards Regression Models for Transition, Lapse in Care, or Death

Care level	Cohort 1: primary care to pulmonary medicine		Cohort 2: pulmonary medicine to lung transplant evaluation		Cohort 3: lung transplant evaluation to waiting list		Cohort 4: waiting list to transplant	
	HR (95% CI)	*P* value	HR (95% CI)	*P* value	HR (95% CI)	*P* value	HR (95% CI)	*P* value
**Transition**								
Diagnosis age (per 5 y)	0.94 (0.94-0.95)	<.001	0.83 (0.82-0.85)	<.001	0.93 (0.91-0.95)	<.001	1.06 (1.03-1.10)	<.001
Male sex	0.98 (0.95-1.01)	.18	2.15 (1.91-2.42)	<.001	1.29 (1.16-1.44)	<.001	1.49 (1.30-1.71)	<.001
Lung disease diagnosis								
Obstructive	1 [Reference]	NA	1 [Reference]	NA	1 [Reference]	NA	1 [Reference]	NA
Restrictive	1.86 (1.74-1.98)	<.001	4.99 (4.44-5.60)	<.001	2.20 (1.96-2.45)	<.001	1.74 (1.51-2.00)	<.001
ADI quintile^a^								
1	1 [Reference]	NA	1 [Reference]	NA	1 [Reference]	NA	1 [Reference]	NA
2	0.91 (0.86-0.96)	.001	1.26 (1.03-1.53)	.02	0.96 (0.81-1.13)	.61	1.12 (0.92-1.36)	.26
3	0.92 (0.87-0.97)	.002	1.42 (1.17-1.71)	<.001	0.84 (0.71-0.99)	.04	1.14 (0.93-1.38)	.20
4	0.90 (0.85-0.95)	<.001	1.44 (1.17-1.77)	<.001	0.76 (0.64-0.92)	.004	1.17 (0.93-1.45)	.18
5	0.87 (0.82-0.92)	<.001	1.69 (1.36-2.09)	<.001	0.55 (0.44-0.68)	<.001	1.20 (0.94-1.54)	.15
Race and ethnicity								
Hispanic	0.98 (0.87-1.10)	.71	0.31 (0.20-0.49)	<.001	NA	NA	NA	NA
Non-Hispanic Black	1.19 (1.15-1.24)	<.001	0.61 (0.51-0.74)	<.001	0.92 (0.75-1.14)	.46	1.21 (0.96-1.53)	.11
Non-Hispanic White	1 [Reference]	NA	1 [Reference]	NA	1 [Reference]	NA	1 [Reference]	NA
**Lapse in care**								
Diagnosis age (per 5 y)	1.00 (0.98-1.01)	.86	0.95 (0.95-0.95)	<.001	NA	NA	NA	NA
Male sex	1.02 (0.95-1.10)	.57	1.00 (0.97-1.02)	.72	NA	NA	NA	NA
Lung disease diagnosis								
Obstructive	1 [Reference]	NA	1 [Reference]	NA	NA	NA	NA	NA
Restrictive	0.70 (0.54-0.91)	.007	1.14 (1.11-1.17)	<.001	NA	NA	NA	NA
ADI quintile^a^								
1	1 [Reference]	NA	1 [Reference]	NA	NA	NA	NA	NA
2	1.18 (1.01-1.38)	.04	0.92 (0.88-0.95)	<.001	NA	NA	NA	NA
3	1.35 (1.16-1.57)	<.001	0.87 (0.84-0.90)	<.001	NA	NA	NA	NA
4	1.47 (1.26-1.72)	<.001	0.84 (0.81-0.88)	<.001	NA	NA	NA	NA
5	1.55 (1.33-1.81)	<.001	0.84 (0.80-0.87)	<.001	NA	NA	NA	NA
Race and ethnicity								
Hispanic	1.05 (0.80-1.38)	.73	1.39 (1.31-1.46)	<.001	NA	NA	NA	NA
Non-Hispanic Black	0.94 (0.85-1.05)	.26	0.94 (0.90-0.97)	<.001	NA	NA	NA	NA
Non-Hispanic White	1 [Reference]	NA	1 [Reference]	NA	NA	NA	NA	NA
**Death**								
Diagnosis age (per 5 y)	1.32 (1.30-1.33)	<.001	1.32 (1.31-1.33)	<.001	1.23 (1.19-1.26)	<.001	1.10 (1.04-1.17)	.001
Male sex	1.29 (1.23-1.36)	<.001	1.48 (1.42-1.54)	<.001	1.29 (1.15-1.45)	<.001	1.17 (0.89-1.54)	.25
Lung disease diagnosis								
Obstructive	1 [Reference]	NA	1 [Reference]	NA	1 [Reference]	NA	1 [Reference]	NA
Restrictive	1.15 (1.02-1.30)	.03	0.98 (0.93-1.03)	.50	1.30 (1.14-1.47)	<.001	1.54 (1.16-2.04)	.003
ADI quintile^a^								
1	1 [Reference]	NA	1 [Reference]	NA	1 [Reference]	NA	1 [Reference]	NA
2	1.23 (1.11-1.35)	<.001	1.25 (1.18-1.34)	<.001	1.08 (0.87-1.34)	.47	1.29 (0.85-1.97)	.23
3	1.46 (1.33-1.61)	<.001	1.48 (1.39-1.58)	<.001	1.07 (0.87-1.31)	.53	1.43 (0.94-2.18)	.10
4	1.75 (1.58-1.92)	<.001	1.59 (1.48-1.70)	<.001	1.46 (1.17-1.81)	<.001	1.40 (0.87-2.26)	.16
5	1.97 (1.78-2.17)	<.001	1.90 (1.77-2.04)	<.001	1.40 (1.11-1.76)	.004	1.97 (1.18-3.29)	.01
Race and ethnicity								
Hispanic	0.85 (0.69-1.04)	.12	0.48 (0.41-0.56)	<.001	NA	NA	NA	NA
Non-Hispanic Black	0.83 (0.77-0.89)	<.001	0.88 (0.82-0.94)	<.001	0.86 (0.69-1.07)	.18	0.35 (0.16-0.75)	.008
Non-Hispanic White	1 [Reference]	NA	1 [Reference]	NA	1 [Reference]	NA	1 [Reference]	NA

Abbreviations: ADI, area deprivation index; NA, not applicable.

^a^ Quintile 1: most resources; quintile 5: least resources.^8^

### Cohort 2: Pulmonary Medicine Patients

There were 73 817 patients who had a pulmonary medicine encounter, and this cohort included individuals who lived out of state and had outside referrals. Of these, 1185 (1.6%) proceeded to lung transplant evaluation, 26 738 lapsed (36.2%), 10 263 died (13.9%), and 35 631 (48.3%) were censored for continuation of encounters to primary care or pulmonary medicine without progressing to lung transplant evaluation (Table 1 and eFigure 1 in Supplement 1). Patients in the least-resourced ADI quintile were 90% more likely to die before reaching a lung transplant evaluation (HR, 1.90 [95% CI, 1.77-2.04]) compared with those in the most-resourced ADI quintile and had a 69% increased likelihood of successfully transitioning to lung transplant evaluation (HR, 1.69 [95% CI, 1.36-2.09]) (Figure 1B and Figure 2B). Black patients experienced a 12% lower likelihood of death (HR, 0.88 [95% CI, 0.82-0.94]) compared with White patients, a 6% lower likelihood of lapse (HR, 0.94 [95% CI, 0.90-0.97]), and a 39% lower likelihood of proceeding to lung transplant evaluation (HR, 0.61 [95% CI, 0.51-0.74]). Hispanic patients experienced a 52% lower likelihood of death (HR, 0.48 [95% CI, 0.41-0.56]) compared with White patients, a 39% higher likelihood of lapse (HR, 1.39 [95% CI, 1.31-1.46]), and a 69% lower likelihood of proceeding to lung transplant evaluation (HR, 0.31 [95% CI, 0.20-0.49]) (Table 2). Those with restrictive lung disease experienced a similar risk of death and a higher risk of lapse (HR, 1.14 [95% CI, 1.11-1.17) and were more likely to proceed to lung transplant evaluation (HR, 4.99 [95% CI, 4.44-5.60]) compared with those with obstructive lung disease. The cumulative incidence of lapse was highest for the youngest patients and for those in the most-resourced ADI quintile (quintile 1), while the risk of death was highest for the oldest patients and for those in the least-resourced ADI quintile (quintile 5) (Figure 1 and eFigure 2B in Supplement 1).

### Cohort 3: Patients Undergoing Lung Transplant Evaluation

**Figure 3.**
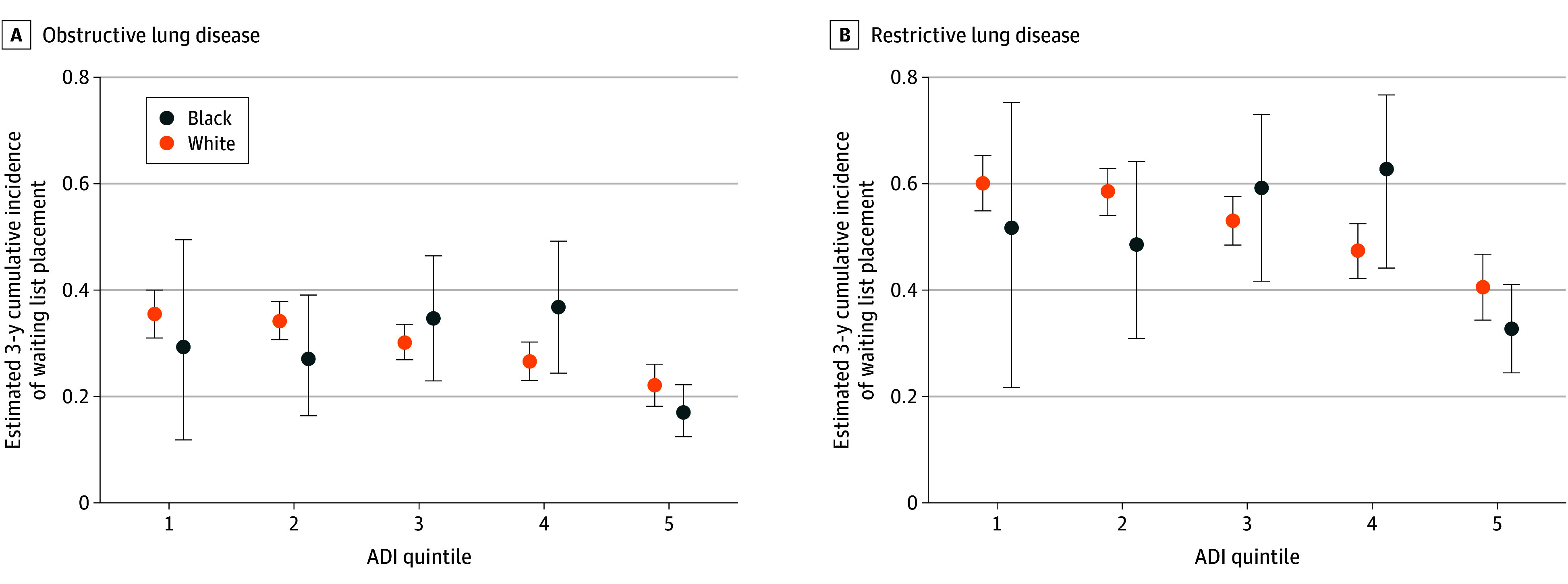
Cumulative Incidence of Waiting List Placement Across Disease Group, Socioeconomic Status, and Black or White Race ADI indicates area deprivation index (quintile 1: most resources; quintile 5: least resources).^8^

Among the patients in this cohort, 4198 were evaluated for lung transplant, 1378 (32.8%) were placed on the lung transplant waiting list, 1120 (26.7%) died, and 1700 (40.5%) were censored (Table 1 and eFigure 1 in Supplement 1). Patients in the least-resourced ADI quintile were 40% more likely to die prior to placement on the waiting list (HR, 1.40 [95% CI, 1.11-1.76]) compared with those in the most-resourced ADI quintile and were 45% less likely to be placed on the waiting list (HR, 0.55 [95% CI, 0.44-0.68]) (Figure 1C and Figure 2C). Black patients experienced similar likelihoods of placement on the waiting list and risk of death compared with White patients. Those with restrictive lung disease were 120% more likely to be placed on the waiting list (HR, 2.20 [95% CI, 1.96-2.45]) and were 30% more likely to die prior to placement on the waiting list (HR, 1.30 [95% CI, 1.14-1.47]) compared with those with obstructive lung disease (Table 2). Racial differences were significantly different across ADI quintiles, although the difference was not consistent over the range of increasing neighborhood deprivation (Figure 3).

### Cohort 4: Patients on the Waiting List

This cohort included 1378 individuals who were placed on the waiting list, 969 (70.3%) who underwent transplant, 222 (16.1%) who died, and 187 (13.6%) who were censored (Table 1 and eFigure 1 in Supplement 1). Patients in the least-resourced ADI quintile were 97% more likely to die prior to transplant (HR, 1.97 [95% CI, 1.18-3.29]) while experiencing a similar rate of transplant compared with patients in the most-resourced ADI quintile (Figure 1D). Black patients were 65% less likely to die prior to transplant (HR, 0.35 [95% CI, 0.16-0.75]) while experiencing a similar likelihood of transplant compared with White patients (Table 2).

## Discussion

In this cohort study, we conceptualized access to transplant as the end result of an accumulating series of care escalations from primary care to transplant. We found that these inequities accumulated across stages of care leading up to wait-listing—steps that to our knowledge have not been studied previously in lung transplantation. We found that patients from the least-resourced ADI quintile were more likely to experience lapses of care early in the care continuum, less likely to reach a waiting list, and more likely to die across all care transitions.

The population of patients evaluated for transplant is subject to survivorship bias.^12^ Patients must survive not only their pulmonary condition but through increasingly specialized and costly levels of care in treating their accumulating morbidity. It is insufficient to only evaluate the transplant-eligible population when studying social disparities in access to transplant.

In 1999, the Institute of Medicine identified gaining access to a waiting list as the primary barrier to accessing transplant for individuals who are socioeconomically disadvantaged.^13^ To address disparate access to transplant, it is essential to first identify points in delivery of care for which inequities arise. This has proven to be difficult in the US lung transplant system due to the lack of a well-established pretransplant registry such as exists in nephrology with the US Renal Data System.^14^ This lack of national data led us to use the EHR of an institution that contains one of the largest US lung transplant centers to develop a blueprint for conducting research on the stages of care that is not available in the US transplant registry.

We found that the probability of successfully transitioning through the care continuum to lung transplant differed across race and ethnicity and SES. Patients in the least-advantaged ADI quintile experienced a lapse in care 55% more often than those in the most-resourced quintile at the level of primary care and a 13% lower transition to pulmonary medicine. Once referred to pulmonary medicine, they were then 69% more likely to undergo a transplant evaluation, yet 45% less likely to be placed on the waiting list. This provides context for the increased risk of death for individuals in the least-resourced ADI quintile across each stage and is consistent with kidney transplantation literature, in which individuals with a lower SES experienced reduced access to organ transplant.^15^ We suspect that the differences in care-continuum progression by SES may be attributable to perceived financial resources, while the overall increased risk of death may be due to differences in disease severity and comorbidity burden. Decisions related to transplant listing are made based on medical need, but a patient’s financial resources (actual and perceived) can impact the referral process.^16^ Care associated with transplant is costly, with estimates of $929 600 to $1 295 900 in the peritransplant period and subsequent medical expenses exceeding $1000 a month.^17,18^ While the OPTN policies clearly state that centers cannot use financial status to discriminate in organ allocation, the financial security requirements for consideration for waitlisting are not explicitly defined.^19^ Given the association between SES and race and ethnicity in the US, limiting access to transplant based on the ability to pay may contribute to unintended and poorly understood structural racism in the transplant system.^16^

We studied the association of race and ethnicity with the ADI with the understanding that the measure of race and ethnicity is intended to capture imposed societal barriers and the contextual patterns of assigned resources and social determinants.^20,21,22^ In this context, we found that Black patients were 19% more likely to transition to pulmonary medicine but were 39% less likely to reach transplant evaluation. However, Black patients experienced similar rates of transition to the waiting list and transplant as did White patients. Despite their lower likelihood of transplant evaluation, Black patients were 12% to 65% less likely to die at each stage. Hispanic patients experienced a similar probability of pulmonary medicine evaluation, were less likely to die prior to transplant evaluation, but were also 69% less likely to reach transplant evaluation. Disparities for minority racial and ethnic groups have been well-described in kidney transplant literature with differences documented in referral practices, time on the waiting list, and transplant rates.^23,24^

Monitoring of the progression of patients through the US transplant system begins once patients are listed for transplant. This structural limitation in the US transplant registry limits the scope of studies of disparities of a population that has already accessed the transplant waiting list. A 2022 National Academies of Sciences, Engineering, and Medicine report recommended that federal oversight should begin earlier than the time of a patient’s placement on the waiting list.^1^ In response, the Human Resources and Services Administration issued a directive to the OPTN in early 2024 to collect data from transplant centers regarding individual transplant referrals, evaluations, and selection processes.^25^ Capturing data even further upstream remains challenging given the heterogeneity in physiologic processes and diseases that result in the final common pathway of end-stage lung disease. This is apparent when assessing the lack of literature addressing the impact of disparities in lung transplant compared with 227 studies for other organs.^26^ Disparities in time to transplant have been demonstrated in the kidney literature, with Black individuals experiencing an increased risk of kidney failure yet being less likely to receive a kidney transplant.^27^ Identification of the population that does not reach lung transplant evaluation is challenging, and its study has only been possible on a large scale for individuals with cystic fibrosis due to the existence of a longitudinal database maintained by the Cystic Fibrosis Foundation.^28,29,30^ The difficulty of capturing upstream data is further compounded by nonintegrated health and health information systems and heterogeneity in lung transplant referral practices and selection criteria. We sought to bridge this knowledge gap by designing this study at a single center, which allowed for a proof of concept to demonstrate how EHR cohort analyses can provide insights into the medical history and care progression for patients with advanced lung disease prior to their arrival on the waiting list. This strategy could present a more cost-effective mechanism to address the Human Resources and Services Administration’s directive to begin systematic data collection in the pretransplant period and extend data capture beyond the point of referral, which may miss patients who were never referred.^25^

### Limitations

Limitations of this study include its necessary single-system design, which may lack external validity outside of large-volume transplant centers. We did not study individuals with pulmonary vascular disease or cystic fibrosis due to small sample sizes and individualized care models that may result in more comprehensive follow-up. The ADI was assigned to individuals based on the census block group of their primary residence, a commonly used method to infer SES, but one that is limited by the risk of ecologic fallacy and missing variation within geographic units.^31,32^ Study of individual socioeconomic factors within given neighborhood environments is an important future direction of study. Finally, inferences for Hispanic patients after wait-listing were limited based on small sample sizes.

## Conclusions

In this cohort study, we characterized access to transplant using an approach that captured the lung transplant continuum of care beginning at primary care and terminating at the time of transplant. We found marked socioeconomic differences in lapses in care and risk of mortality in transitioning to pulmonary medicine and to transplant medicine, suggesting multiple potential points of intervention for advancing equitable access to lung transplant.

## Notes

Supplement 1. **eFigure 1.** Patient Flowchart **eFigure 2.** Conditional Probability of Lapse From Primary Care to Pulmonary Medicine and Pulmonary Medicine to Lung Transplant Evaluation **eTable 1.** Diagnosis Code Inclusion and Diagnosis Group Assignment **eTable 2.** Department Specialty and Transition Assignment **eTable 3.** UNOS Data Elements **eFigure 3.** Cumulative Incidence of Death Across the Lung Transplant Care Continuum by Socioeconomic Status **eFigure 4.** Conditional Probability of Transition Across the Lung Transplant Care Continuum by Socioeconomic Status **eFigure 5.** Conditional Probability of Lapse From Primary Care to Pulmonary Medicine and Pulmonary Medicine to Lung Transplant Evaluation

Supplement 2.
Data Sharing Statement
