# Supplementary material for: Socioeconomic Differences in Navigating Access to Lung Transplant
Source: JAMA Netw Open. 2025 Mar 13;8(3):e250572. doi: 10.1001/jamanetworkopen.2025.0572 (PMC11907320; doi:10.1001/jamanetworkopen.2025.0572)
Supplement: Supplement 1. — eFigure 1. Patient Flowchart eFigure 2. Conditional Probability of Lapse From Primary Care to Pulmonary Medicine and Pulmonary Medicine to Lung Transplant Evaluation eTable 1. Diagnosis Code Inclusion and Diagnosis Group Assignment eTable 2. Department Specialty and Transition Assignment eTable 3. UNOS Data Elements eFigure 3. Cumulative Incidence of Death Across the Lung Transplant Care Continuum by Socioeconomic Status eFigure 4. Conditional Probability of Transition Across the Lung Transplant Care Continuum by Socioeconomic Status eFigure 5. Conditional Probability of Lapse From Primary Care to Pulmonary Medicine and Pulmonary Medicine to Lung Transplant Evaluation [file jamanetwopen-e250572-s001.pdf]

Supplemental Online Content

Lehr CJ, Mourany L, Gunsalus P, Rose J, Valapour M, Dalton JE. Socioeconomic differences in navigating access to lung transplant. *JAMA Netw Open*. 2025;8(3):e250572. doi:10.1001/jamanetworkopen.2025.0572

eFigure 1. Patient Flowchart
eFigure 2. Conditional Probability of Lapse From Primary Care to Pulmonary Medicine and Pulmonary Medicine to Lung Transplant Evaluation
eTable 1. Diagnosis Code Inclusion and Diagnosis Group Assignment
eTable 2. Department Specialty and Transition Assignment
eTable 3. UNOS Data Elements
eFigure 3. Cumulative Incidence of Death Across the Lung Transplant Care Continuum by Socioeconomic Status
eFigure 4. Conditional Probability of Transition Across the Lung Transplant Care Continuum by Socioeconomic Status
eFigure 5. Conditional Probability of Lapse From Primary Care to Pulmonary Medicine and Pulmonary Medicine to Lung Transplant Evaluation

This supplemental material has been provided by the authors to give readers additional information about their work.

© 2025 Lehr CJ et al. *JAMA Network Open*.

eFigure 1. Patient Flowchart

Figure depicts number of patients with obstructive and restrictive lung disease in each state. Black arrows indicate number of deaths, red arrows indicate lapse in care of over 2 years (or not being waitlisted), and gray indicate censored patients. Each row of boxes represents different populations to maximize data capture at each level with curved arrows demonstrating the additional sources of data incorporated between levels. Each black horizontal arrow represents one of the transitions studied: 1) primary care to pulmonary medicine, 2) pulmonary medicine to lung transplant evaluation, 3) lung transplant evaluation to placement on the waiting list, 4) waiting list placement to transplant.

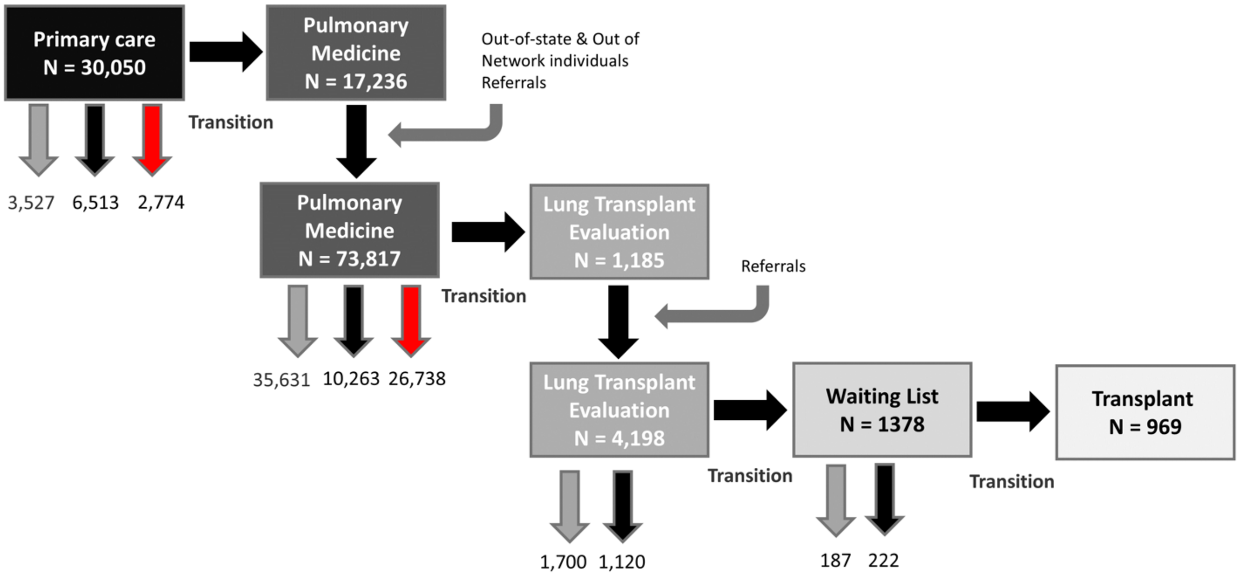

© 2025 Lehr CJ et al. *JAMA Network Open*.

eFigure 2. Conditional Probability of Lapse From Primary Care to Pulmonary Medicine and Pulmonary Medicine to Lung Transplant Evaluation

A) 3-year conditional probability of care lapse from primary care to pulmonary medicine given that they did not die by age and ADI, for a male patient with restrictive lung disease

B) 3-year cumulative incidence of care lapse from pulmonary medicine to lung transplant evaluation by diagnosis group, age, and ADI. Displayed are predictions for a male patient with restrictive lung disease.

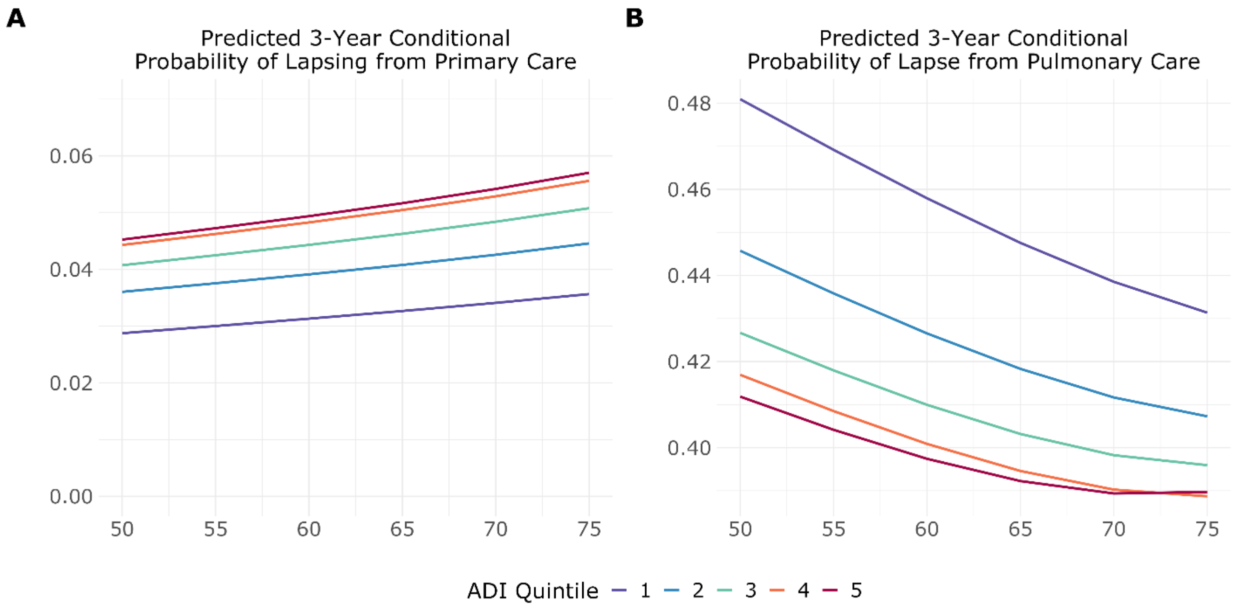

© 2025 Lehr CJ et al. *JAMA Network Open*.

eTable 1. Diagnosis Code Inclusion and Diagnosis Group Assignment

ConceptID	Diagnosis Group	Description	ICD9 Code	ICD10 Code
C0152586	A	Tuberculous bronchiectasis	011.50	A15.0
C0152587	A	Tuberculous bronchiectasis, bacteriological or histological examination not done	011.51	
C0152588	A	Tuberculous bronchiectasis, bacteriological or histological examination unknown (at present)	011.52	
C0152590	A	Tuberculous bronchiectasis, tubercle bacilli not found (in sputum) by microscopy, but found by bacterial culture	011.54	
C0152591	A	Tuberculous bronchiectasis, tubercle bacilli not found by bacteriological examination, but tuberculosis confirmed histologically	011.55	
C0152592	A	Tuberculous bronchiectasis, tubercle bacilli not found by bacteriological or histological examination, but tuberculosis confirmed by other methods [inoculation of animals]	011.56	
C0155872	A	Catarrhal bronchitis	491.0	J41.0
C0155873	A	Mucopurulent chronic bronchitis	491.1	J41.1
C0155874	A	Emphysematous bronchitis	491.2	J44
C0155875	A	Obstructive chronic bronchitis without exacerbation	491.20	
C4041147	A	Acute exacerbation of chronic obstructive bronchitis	491.21	
C1456131	A	obstructive chronic bronchitis with acute bronchitis	491.22	
C0029544	A	Other chronic bronchitis	491.8	
C0008677	A	Bronchitis, Chronic	491.9	J42
C0034067	A	Pulmonary Emphysema	492	J43.9
C0152242	A	Emphysematous bleb	492.0	J43.9
C0029607	A	Other emphysema	492.8	J43.8
C0155883	A	Chronic obstructive asthma (with obstructive pulmonary disease)	493.2	J44
C0375333	A	Chronic obstructive asthma, unspecified	493.20	
C0375334	A	chronic obstructive asthma with status asthmaticus	493.21	
C1176341	A	chronic obstructive asthma with acute exacerbation	493.22	
C0006267	A	Bronchiectasis	494	J47.9
C0878695	A	bronchiectasis without acute exacerbation	494.0	
C0302378	A	Chronic airway obstruction, NEC in ICD9CM_2013_2012_08_06	496	
C0751674	A	Lymphangioleiomyomatosis	516.4	J84.81
C1370824	A	Interstitial emphysema	518.1	J98.2
C0155918	A	Compensatory emphysema	518.2	J98.3
C0152239	A	Congenital bronchiectasis	748.61	Q33.4
C0158936	A	Interstitial emphysema and related conditions	770.2	
C0348807	A	Mixed simple and mucopurulent chronic bronchitis		J41.8
C2887442	A	Unilateral pulmonary emphysema [MacLeod's syndrome]		J43.0
C0264393	A	Panacinar Emphysema		J43.1
C0221227	A	Centriacinar Emphysema		J43.2
C0348818	A	Chronic obstructive pulmonary disease with acute lower respiratory infection		J44.0

© 2025 Lehr CJ et al. *JAMA Network Open*.

C0340044	A	Acute exacerbation of chronic obstructive airways disease		J44.1
C0024117	A	Chronic Obstructive Airway Disease		J44.9
C2887468	A	Bronchiectasis with acute lower respiratory infection		J47.0
C2887469	A	uncomplicated bronchiectasis		J47.9
C0175999	D	Post-inflammatory pulmonary fibrosis	515	J84.10
C0034050	D	Pulmonary Alveolar Proteinosis	516.0	J84.01
C0020807	D	Idiopathic pulmonary hemosiderosis	516.1	J84.03
C0155912	D	Pulmonary Alveolar Microlithiasis	516.2	J84.02
C3161100	D	Idiopathic interstitial pneumonia, not otherwise specified	516.30	J84.111
C1800706	D	Idiopathic Pulmonary Fibrosis	516.31	J84.112
C3161102	D	Idiopathic non-specific interstitial pneumonitis	516.32	J84.113
C1279945	D	Acute interstitial pneumonia	516.33	J84.114
C0242770	D	Bronchiolitis Obliterans Organizing Pneumonia	516.36	J84.116
C0238378	D	Desquamative interstitial pneumonia	516.37	J84.115
C3161104	D	Adult pulmonary Langerhans cell histiocytosis	516.5	J84.82
C3161105	D	Neuroendocrine cell hyperplasia of infancy	516.61	J84.841
C3161106	D	pulmonary interstitial glycogenosis	516.62	J84.842
C3161107	D	surfactant mutations of lung	516.63	J84.83
C3161109	D	Other interstitial lung diseases of childhood	516.69	J84.848
C3264392	D	Other alveolar and parieto-alveolar conditions		J84.09
C3264396	D	Pulmonary fibrosis, unspecified		J84.10
C3264401	D	Other interstitial pulmonary diseases with fibrosis in diseases classified elsewhere		J84.17
C5384725	D	interstitial lung disease with progressive fibrotic phenotype in diseases classified elsewhere		J84.170
C0264511	D	Lymphoid interstitial pneumonia		J84.2
C0348704	D	Other specified interstitial pulmonary diseases		J84.89
C0206062	D	Lung Diseases, Interstitial		J84.9

© 2025 Lehr CJ et al. *JAMA Network Open*.

eTable 2. Department Specialty and Transition Assignment

Transition Stage	Department/Specialty	Any coding info needed?
**Primary Care**	Internal Medicine<br>Family Medicine<br>Emergency Medicine<br>OBGYN<br>Geriatrics	Department specialty<br>Department specialty<br>Department specialty<br>Department specialty<br>Department specialty
**Pulmonary Medicine**	Pulmonology<br><br>Pediatric Pulmonology	Department specialty<br><br>Department specialty
**Lung Transplant**	Pulmonary Transplant	Department Name
**Waiting List**	UNOS listing data obtained from EHR	Chart review by transplant team
**Transplant**	UNOS listing data obtained from EHR	Chart review by transplant team

© 2025 Lehr CJ et al. *JAMA Network Open*.

eTable 3. UNOS Data Elements

CAS Score at Time of Listing
LAS Score at Time of Listing
Initial LAS Score at Time of Match
LAS Score at Time of Removal
CAS Score at Time of Removal
Removal LAS Score at Time of Match
Listing Date
Age at time of Match Run
Current Bilirubin
BMI
Cardiac Index
Assisted Ventilation
Diagnosis Group
Diagnosis
Current Creatinine
Functional Status
Supplemental Oxygen Amount
pCO2 mmHg
Pulmonary Artery Systolic Pressure
Six Minute Walk Distance

© 2025 Lehr CJ et al. *JAMA Network Open*.

eFigure 3. Cumulative Incidence of Death Across the Lung Transplant Care Continuum by Socioeconomic Status

Model-predicted 3-year cumulative incidence of death prior to transitioning to the next level of care (cohort 1: primary care to pulmonary medicine (**Panel A**); cohort 2: pulmonary medicine to lung transplant evaluation (**Panel B**); cohort 3: lung transplant evaluation to waiting list (**Panel C**)). Model-predicted 1-year cumulative incidence of death prior to transitioning to lung transplant (cohort 4: waiting list to lung transplant (**Panel D**)), across CCHS patients grouped according to Area Deprivation Index (ADI) quintiles associated with place of residence. Estimates are provided across age and ADI for a male patient with *obstructive lung disease*.

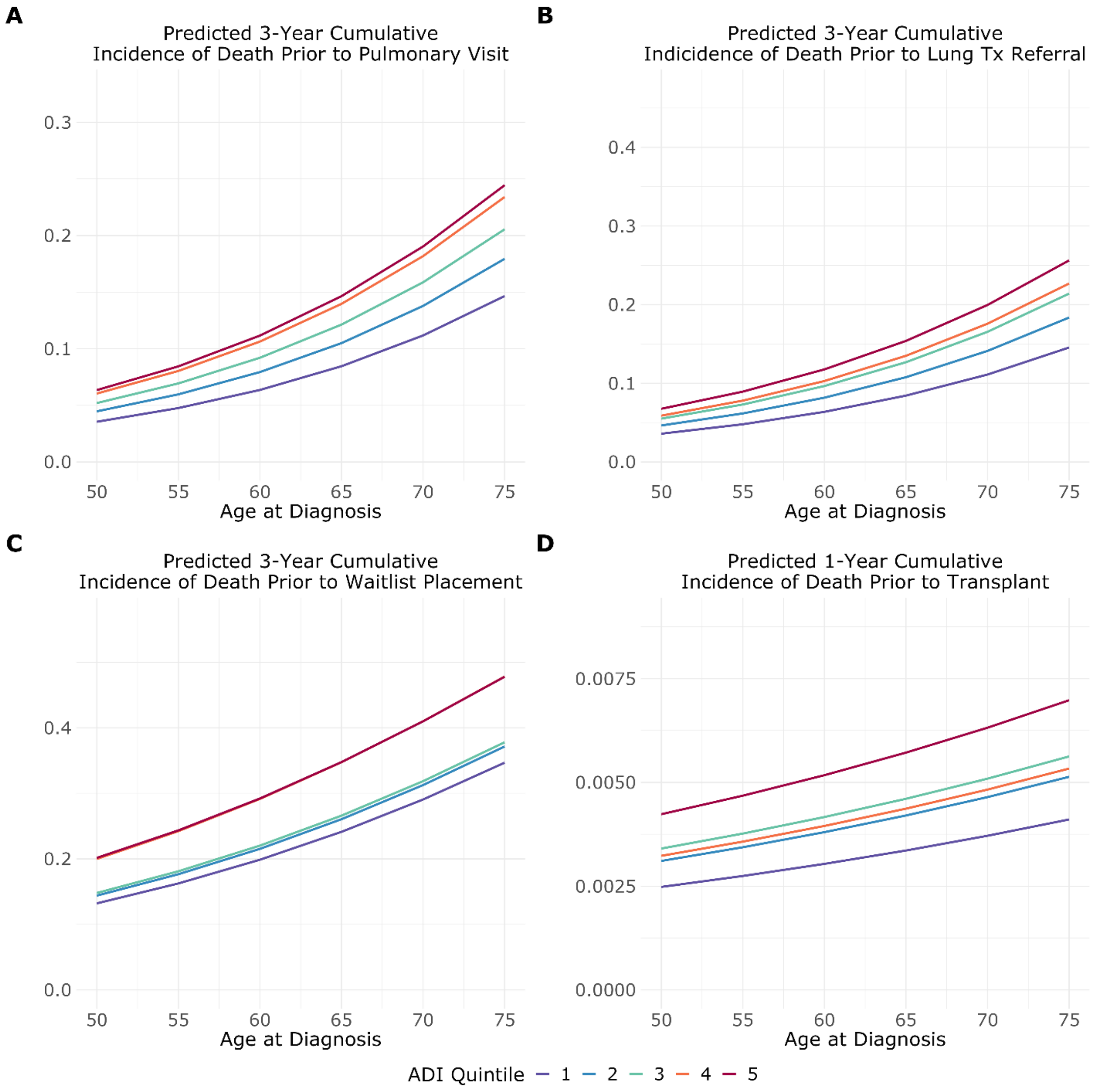

© 2025 Lehr CJ et al. *JAMA Network Open*.

eFigure 4. Conditional Probability of Transition Across the Lung Transplant Care Continuum by Socioeconomic Status

3-year conditional probability of transition between A) primary care to pulmonary medicine (cohort 1), B) pulmonary medicine to lung transplant evaluation (cohort 2), C) lung transplant evaluation to waiting list (cohort 3). 1-year conditional probability of transition from D) waiting list to lung transplant (cohort 4). Estimates are provided across age and ADI and report the probability of transition assuming patients did not die during the period for a male patient with *obstructive lung disease*.

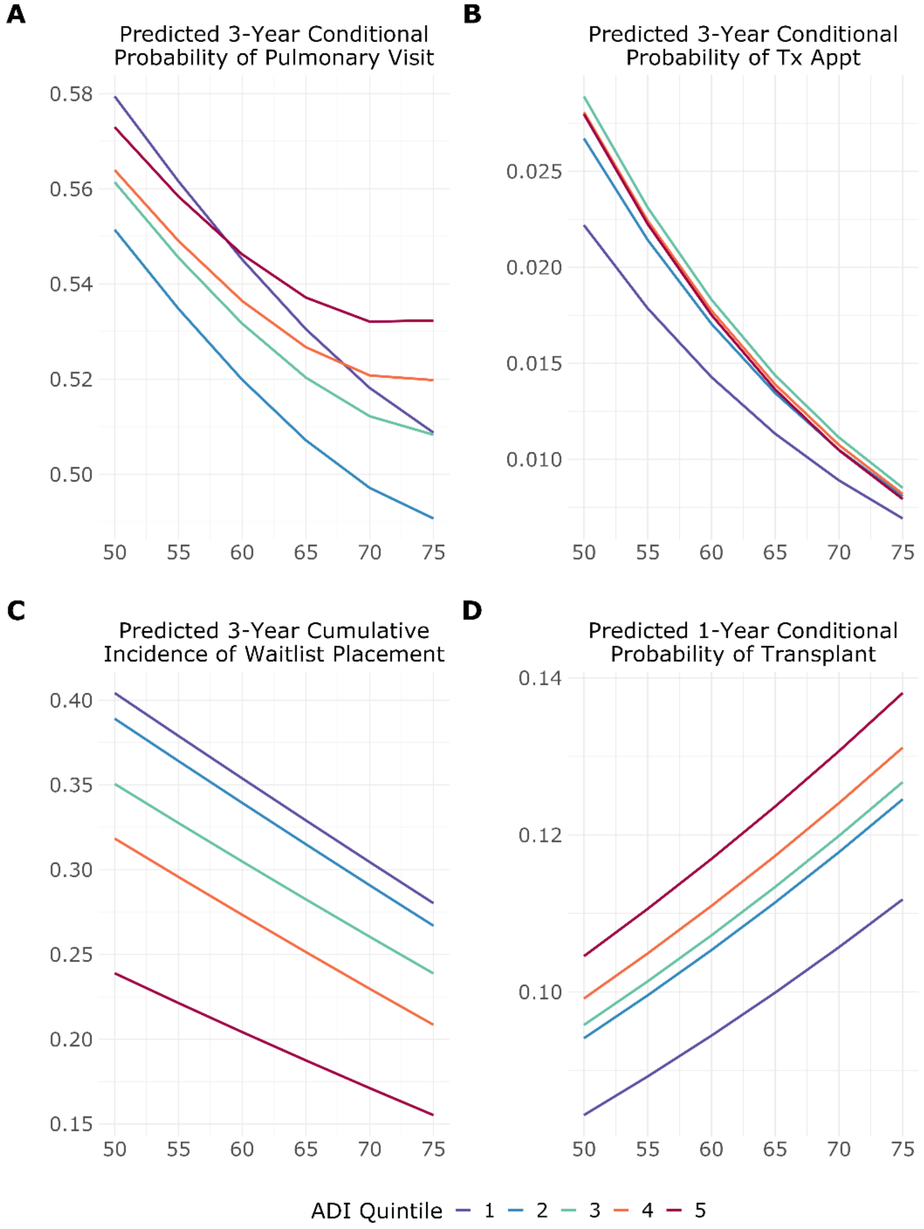

© 2025 Lehr CJ et al. *JAMA Network Open*.

eFigure 5. Conditional Probability of Lapse From Primary Care to Pulmonary Medicine and Pulmonary Medicine to Lung Transplant Evaluation

A) 3-year conditional probability of care lapse from primary care to pulmonary medicine given that they did not die by age and ADI, for a male patient with obstructive lung disease

B) 3-year cumulative incidence of care lapse from pulmonary medicine to lung transplant evaluation by diagnosis group, age, and ADI. Displayed are predictions for a male patient with *obstructive lung disease*.

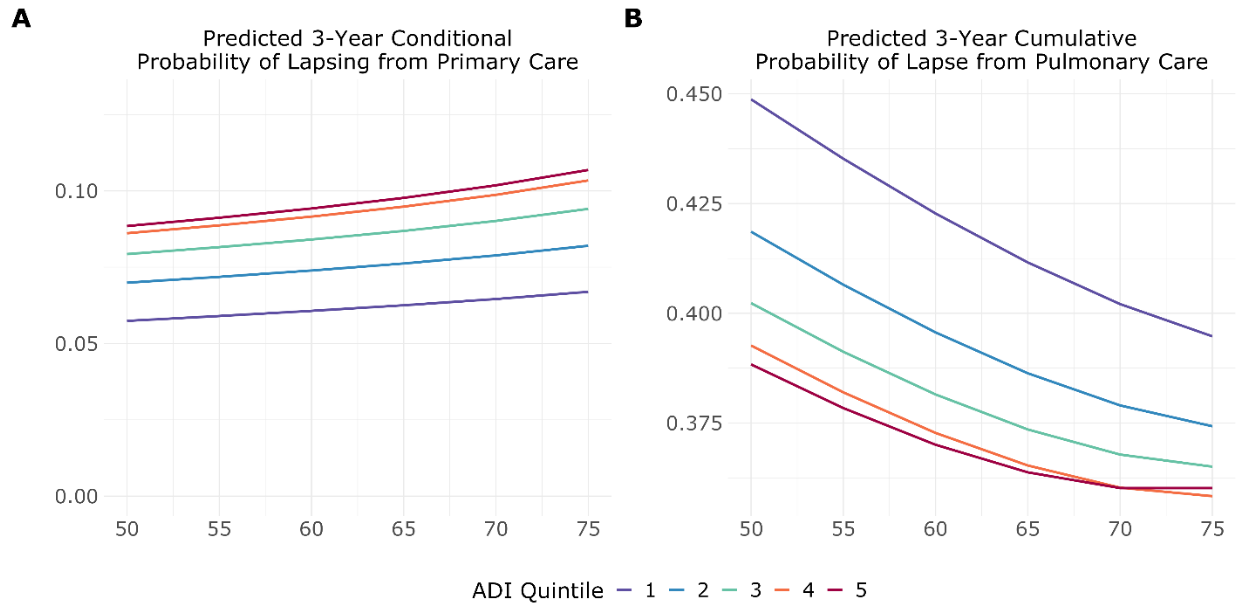

© 2025 Lehr CJ et al. *JAMA Network Open*.
