# Supplementary material for: Socioeconomic Differences in Navigating Access to Lung Transplant
Source: JAMA Netw Open. 2025 Mar 13;8(3):e250572. doi: 10.1001/jamanetworkopen.2025.0572 (PMC11907320; doi:10.1001/jamanetworkopen.2025.0572)
Supplement: Supplement 2. — Data Sharing Statement [file jamanetwopen-e250572-s002.pdf]

Data Sharing Statement

Lehr. Socioeconomic Differences in Navigating Access to Lung Transplant. *JAMA Netw Open*. Published March 13, 2025. doi:10.1001/jamanetworkopen.2025.0572

Data
Data available: No

Additional Information
Explanation for why data not available: PHI in dataset
